# Hypoxia-Inducible Factor Is Critical for Pathogenesis and Regulation of Immune Cell Functions in Rheumatoid Arthritis

**DOI:** 10.3389/fimmu.2020.01668

**Published:** 2020-07-28

**Authors:** Xin Guo, Guangjie Chen

**Affiliations:** Department of Immunology and Microbiology, Shanghai JiaoTong University School of Medicine, Shanghai Institute of Immunology, Shanghai, China

**Keywords:** hypoxia, hypoxia-inducible factor, rheumatoid arthritis, inflammation, pannus formation, cartilage destruction, bone erosion

## Abstract

Rheumatoid arthritis (RA) is a common autoimmune disease with characteristics of synovial inflammation, pannus formation, cartilage destruction, and bone erosion. Further, the inflammation is linked to increased oxygen consumption, resulting in hypoxia within the inflammatory area. Hypoxia-inducible factor (HIF) was reported to be associated with adaptation to the hypoxic microenvironment in the RA synovium. Here, we have briefly summarized the structure and expression of HIF. Moreover, the function of HIF in inflammation, angiogenesis, cartilage damage, and immune cells of RA has been discussed.

## Introduction

Rheumatoid arthritis (RA) is the most common chronic inflammatory disease, with characteristics of synovial inflammation, pannus formation, cartilage destruction, and bone erosion, which ultimately cause deformity of the affected joints (1). While the etiology and pathogenesis of RA have not been clearly elaborated, studies have shown that both environmental and genetic factors are involved in its etiology (2). Additionally, different types of immune cells have been shown to be involved, including macrophages, dendritic cells, T cells, B cells, neutrophils, and mast cells. Apart from the complex interactions between immune cells in such area, the microenvironment of synovial fluid is composite in which newly-formed highly dysfunctional straight and regularly-branching vessels resulting in hypoxia and reduced oxygen supply. According to recent studies, oxygen tension is associated with cell proliferation, division, and survival, which is considered relevant to the pathogenesis of RA (2). In particular, synovial hypoxia, defined as low oxygen tension in the synovium, is a potential pathogenetic factor and plays a crucial role in promoting angiogenesis as well as the pathophysiological response in RA (3, 4). In response to the alterations of oxygen tension in the inflamed joint micro-environment, hypoxia-inducible factors (HIFs) are activated and overexpressed to regulate the transcription and expression of genes related to inflammation, angiogenesis, energy metabolism, and other processes (4). Moreover, HIF is a type of nuclear transcription factor that can stimulate angiogenesis, promote pannus formation, and aggravate synovial hyperplasia (5, 6). Additionally, recent studies have shown that HIF plays an important role in adaptation to hypoxic environments.

## Structure and Activation of Hypoxia-Inducible Factors (HIFS)

HIF is a heterodimeric complex, consisting of α subunits (HIF-1α, HIF-2α, or HIF-3α) and a β subunit (also known as aryl hydrocarbon receptor nuclear translocator or ARNT). Expression of the α subunit is regulated by oxygen concentration in the cytoplasm, while the β subunit is constitutively expressed in the cell nucleus (7, 8). Both α and β subunits, which contain a basic helix-loop-helix (bHLH) domain and a PAS-A and PAS-B (Per, Arnt, and Sim; PAS) domain, could combine as a heterodimeric complex to bind to the hypoxia response element (HRE) within specific sequences of the promotors of target genes. Under physiological tissue oxygen tensions, HIF-1α is targeted for degradation by hydroxylation of specific prolyl residues (Pro402 and Pro564) within the ODD domain, which is catalyzed by specific enzymes prolyhydroxylases (PHDs), accompanied by ferrous iron (Fe^2+^) as an enzymatic cofactor. PHDs are active when oxygen is available, targeting HIF-1α for proteasomal degradation via the Von Hippel-Lindau tumor suppressor protein (pVHL)-dependent ubiquitination, however, their activity declines in hypoxic conditions (9, 10). Apart from O_2_ and Fe^2+^ serving as cofactors, both degradation pathways require a-ketoglutarate as a co-substrate (11).

Noteworthy, the expression of HIF-1α is up-regulated during an initial response (<24 h) of intense hypoxia or anoxia (<0.1% O_2_), whereas HIF-2α and HIF-3α are overexpressed in chronic hypoxic situations (>24 h) of mild or physiological hypoxia (<5% O_2_) (12, 13).

Under hypoxia conditions, posttranslational hydroxylation modification of these two proteins is inhibited, resulting in stabilization of the HIF-α levels due to the decreased activity of PHDs and FIH, leading to a low affinity between HIF-α and pVHL (14). As for the activity of FIH and PHD, a survey suggests that the PHD inhibitors may only partially up-regulate the HIF transcriptional response, and the biochemical analysis revealed that FIH activity is inhibited at lower oxygen tension than PHD. Thus, PHD activity may reduce first with decreases in oxygen levels, leading to the accumulation of HIF-α in the cytoplasm (15).

Oxygen-independent factors may also induce and activate HIF-α. Additionally, heat, low pH, and biochemical factors such as cytokines, growth factors, and reactive oxygen species (ROS), may play a vital role in the induction and activation of HIF. Further, it has been reported that bacterial lipopolysaccharides may have the ability to induce HIF-1α in human macrophages and monocytes via nuclear factor-kappa B (NF-κB) and p44/42 mitogen-activated protein kinase (MAPK) pathways (4). Moreover, the accumulation of tumor necrosis factor-α (TNF-α) within the injury area has been shown to promote HIF-1α accumulation in initial inflammatory cells, with no changes in its transcription level.

## Role of Hif in Ra Pathogenesis

### HIF Expression in RA Joints

HIFs are more highly expressed in the hyperplastic RA synovium, which mainly include macrophage-like synoviocytes (MLS) and fibroblast-like synoviocytes (FLS) (16, 17), than that in osteoarthritis (OA) patients (18). However, the degree of different HIF isoforms expression has begun to be elucidated. Of note, HIF-1α is reported to be strongly expressed in the intimal layer of the RA synovium, including in resident macrophages (6). By contrast, some studies showed that the expression of HIF-1α is sparse, while HIF-2α is the predominant isoform in both human RA synovium and the collagen-induced arthritis model (CIA). Further, the expression of HIF-2α is mainly observed on FLS of the RA synovium (19). Moreover, HIF-1α and HIF-2α are expressed in resident and infiltrating immune cells, as well as chondrocytes and osteoclasts (19).

### Role of HIF in Inflammation

**Figure 1:**
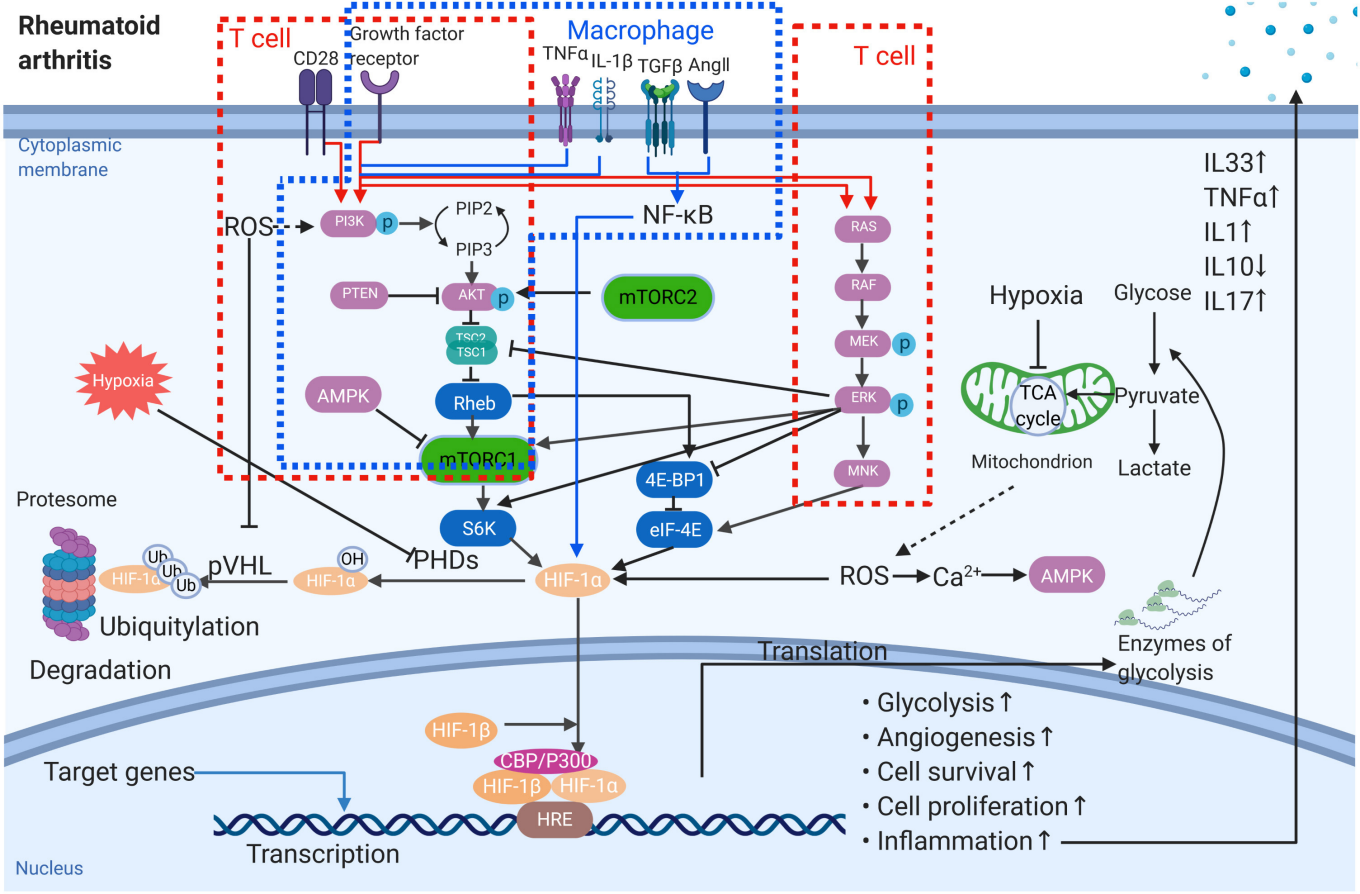
The role of HIF-1α in the pathogenesis of rheumatoid arthritis. Multiple signals affect the protein amount of HIF-1α, the activation of HIF-1α as well as the target gene expression. The red frames stand for T cells while the blue one is macrophage. → stimulation; ⊣ inhibition.

An increasing number of reports has revealed that HIFs act as key regulators in RA inflammation, as they can mediate this inflammation by different aspects (Figure 1). For example, hypoxia-related epithelial-mesenchymal transition (EMT) has been observed in the FLS through the PI3 kinase/Akt/HIF-1 pathway (20). In particular, one key inflammatory cascade, TNF, is overexpressed (10). Toll-like receptors (TLR), the typical recognition receptors, are also mainly observed in immune cells and rheumatoid arthritis synovial fibroblasts (RASF) in RA to regulate the inflammatory response (21, 22). Kim et al. stated that the HIF-1α-related pathway was able to up-regulate TLR-4 expression in macrophages, activating inflammation. Further, TNF-α converting enzyme, TNF-α release, and TLR are all HIF-1-dependent processes in RA disease. As pro-inflammatory factors, TNF-α, interleukin 1 (IL-1), and IL-33 production are increased in affected synovial fluid and tissue. Thus, TNF and IL-1 may function synergistically to induce effector functions. Additionally, IL-33 levels are increased in RA patients, showing a role of this inflammatory factor in the severity of RA (23). As previously shown, TNF-α is a key factor that promotes HIF accumulation. Further, IL-1 and IL-33 have been shown to increase the expression of HIF isoforms in synovial fibroblasts (19, 24). Accordingly, the ability of the HIF-1α/IL-33 regulatory circuit to further increase HIF-1α is formed by the enhanced expression of IL-33 correlated with HIF-1α up-regulation. Hu et al. (25) demonstrated that HIF-1α overexpression was likely to stimulate inflammatory cytokine expression in polyIC-stimulated RASF, leading to the shift toward a pro-inflammatory state in RA. Besides its pro-inflammatory function, HIFs can activate production of anti-inflammatory cytokines, such as IL-10. Meng et al. showed that mice with a specific HIF-1α deletion displayed a reduced number of IL-10 production B cells, followed by enhanced Th17 cells, which ultimately exacerbated the collagen-induced arthritis (CIA).

### Role of HIF in Angiogenesis

In RA, the sub-intimal layer of the synovium is heavily infiltrated by immune cells and undergoes neovascularization. Neovascularization is generally connected to hypoxia in both physiological and pathological states (26, 27). Angiogenesis, a complex process of new blood-vessel formation as well as expression of multiple genes, is a typical characteristic of RA and likely a consequence of hypoxia in affected joints (10). Numerous genes have been shown to be involved during different steps of angiogenesis by hypoxia challenge. Further, expansion of the synovial tissue is essential for the newly-generated vessels to supply oxygen and adequate nutrients in the hypertrophic synovium. However, the oxygen supply via dysregulated vasculature is inadequate. Moreover, ROS generation may further enhance damage within joints. Fava et al. (28) and Koch et al. (29) demonstrated that HIFs were capable of regulating the expression of proangiogenic mediators, including chemokine IL-8 (CXCL8), CC-chemokine ligand 20 (CCL20, known as macrophage inflammatory protein 3α, MIP-3α), and vascular endothelial growth factor (VEGF) (30). Among these, VEGF, the prime angiogenesis target of HIF-1α with hypoxia-dependent expression, acts as the most potent endothelial-specific mitogen in RA. VEGF is a cytokine that acts on the vascular endothelium of the synovium, promoting angiogenesis and binding to cognate receptors on endothelial cells (ECs), which activates these cells to produce more proteolytic enzymes. Compared with normal synovium, angiopoietin (Ang)1, Ang2 as well as tyrosine kinase receptor (Tie)2 are also highly elevated in RA. With the model of adjuvant arthritis, the upregulation of PI3K, Akt1, p-Akt1, and mammalian target of rapamycin (mTOR) indicate that the activation of PI3K/Akt/ mTOR signaling may participants in the induction of new-formed highly dysfunctional synovial blood vessels (Figure 1). Further, inhibition of HIF-1 expression could significantly reduce the VEGF-induced angiogenesis in FLS of RA (28). In RA synovial tissue, inflammation contributes to the hypoxic environment, and HIF-1α is dramatically increased to promote cells to be more tolerant of low oxygen tension (31). Simultaneously, the expression of HIF-1α and VEGF in synovial tissue is influenced by angiogenesis (31), and HIF-1α and HIF-2α isoforms are expressed in the RA synovium at levels related to the magnitude of the angiogenetic response (32).

### Role of HIF in Cartilage Damage

Articular cartilage destruction and bone erosion are vital features of RA disease. With the progression of inflammation, hyperplasia pannus invades and destroys the cartilage of RA-related joints. HIF is a key factor for cell adaptation to hypoxia in RA. The activation of HIF-1α in cartilage maturation has been demonstrated as necessary for chondrocytes survival and homeostasis. Notably, HIF-1α is a key component that regulates the inhibition of cartilage hypertrophy and the maintenance of chondrogenic specific markers, such as aggrecan, SOX9. The dysregulation of HIF-1α would lead to skeletal dysplasia. *In vivo* experiment showed that expression of HIF-1α suppresses NF- κB—HIF-2α signaling, which is the potent upstream pathway of MMP13. Proteinases, such as matrix metalloproteinases (MMPs) and ADAMTS, known as a disintegrin and metalloproteinase with thrombospondin motifs, can cause a direct damage to the cartilage. The family of MMP includes cartilage-degrading enzymes produced by the type B synoviocytes in RA. Expression of MMPs is elevated during the repair and remodeling of damage tissues. Moreover, tissue inhibitors of metalloproteinases (TIMPs), serine proteinase inhibitors (SERPINS), and α2-macroglobutin can regulate the activity of MMPs (33). Accompanied by HIF-1, the expression of the IL-1β-stimulating-MMP1/MMP13 and the IL-17/TNF-α-promoting -MMP2/MMP9 result in the greater migration and invasive ability of RA FLS (34).

Due to activation of osteoclasts under hypoxia, articular cartilage destruction is exacerbated. Swales et al. (35) reported that angiopoietin-like 4 (ANGPTL4) is overexpressed in RA osteoclasts in a HIF-1α-dependent manner, stimulating bone resorption mediated by osteoclasts.

## Effect of HIF on Immune Cells in RA

### HIF and T Cells

Makino et al. (36) proved that low oxygen tension is able to promote the survival of T cells by stabilizing HIF-1α. T cells have been hypothesized to be involved in disease pathogenesis. Apart from circulating in the bloodstream, they exist in areas of oxygen tension, as low as 0.5%. Foxp3 is the typical marker of regulatory T cells, and previous studies have proven that hypoxia is a factor that increases Foxp3 expression on T cells, whereas HIF-1α functions conversely compared to the low oxygen levels (37). Except for Treg cells, evidence has emerged to identify that other T-cell subsets with various functions exist, including Th1, Th2, Th17, and Tfh cells (38). For example, overexpression of HIF-1 enhances the RA synovial fibroblast-mediated expansion of inflammatory Th1 and Th17 cells. Within the inflamed joint of RA, low oxygen level is responsible for the upregulation of HIF-1 in naïve CD4^+^ T cells. Moreover, metabolic reprogramming in T cells can be induced by low oxygen levels. HIF-1 is able to activate the expression of Th17 key regulator RORγt by mTOR signaling, while HIF-1 downregulate Foxp3 protein levels. It is believed that the upregulation of HIF-1 has a greater impact on Th17 differentiation. However, the role of HIF-1 in Th17/ Treg balance remains to be established. mTOR acts as an important sensor for the T-cell response as well as interactions with other metabolic pathways. Also, mTOR spurs on glycolysis via the upregulation of HIF-1 (39) (Figure 1). Further, Foxp3+ Treg cells have been shown to perform converse functions to Th17 cells, which may be correlated with the hypoxic environment in RA. Other studies also highlighted that differentiation to Th17 cells was associated with the expression of the HIF-1α-driven glycolytic genes (40).

### HIF and Macrophages

Extensive studies of innate and adaptive immune cells, in which the role of monocyte and macrophages are all discussed, have been done. Monocytes are essential in the initiation and maintenance of synovial inflammation in RA. Circulated monocytes are recruited to the RA synovium via chemotaxis interacting with chemotactic ligands presented by other autoimmune cells within the synovium. On the one hand, monocytes can differentiate into specific pro-inflammatory subsets. On the other hand, it is capable to differentiate into macrophages to promote synovial inflammation. Amp-activated protein kinase (AMPK) is an energy-sensing enzyme in macrophages. It counters metabolic changes induced in pro-inflammatory macrophages by stabilizes the inhibition of IκBα, which antagonizes nuclear factor κB (NF-κB) signaling. In particular, AMPK activity is high in M2 macrophages which is able to drive the production of anti-inflammatory cytokines. Under hypoxia microenvironment, the expression of HIF-1α is upregulated in M1 macrophages rather than M2, regulating the metabolic switch. Macrophages are considered key inflammatory factors within the synovium in RA. As is well-known, the oxygen tension of RA patients is equivalent to 2–4%, representing the hypoxic microenvironment. There are two types of macrophages: M1 and M2, for which M1 macrophages are able to kill intracellular microorganisms, while M2 macrophages exert anti-inflammatory effects (41). M1 phenotypes are associated strongly with tumors. Conversely, knockout of HIF-1α skewed cells toward a more M2 phenotype, accompanied by HIF-1 and HIF-2 in the hypoxic response. As well, increasing studies have proven HIF-2α to be the predominant isoform in this process. Under hypoxia, oxidative phosphorylation is inhibited, which causes macrophages to shift to glycolysis to produce ATP. HIF-1α is able to regulate the expression of the transcriptional glycolysis-related enzymes and inhibit oxidative phosphorylation. Noteworthy, macrophages activate HIF-1α to promote the production of inflammatory factors (Figure 1), while avoiding excessive inflammatory activation by inhibiting NF-κB.

### HIF and Other Myeloid Cells

In terms of other myeloid cells, HIF is involved in the regulation of neutrophil apoptosis. Mecklenburgh et al. (42) inhibited the HIF-1α degradation pathway using an ion chelator and found that hypoxia regulated the NF-κB pathway in a HIF-1α-dependent manner. Further, hypoxia was found to promote neutrophil secretion of MIP-β (Macrophage inflammatory protein-1β), leading to neutrophil survival and continued secretion of inflammatory factors.

Under hypoxia conditions, the expression of over 2,000 genes was shown to be induced during monocyte differentiation into dendritic cells (DCs) (38). Conventionally, DCs are antigen-presenting cells (APC), connecting innate and adaptive immunity. However, under hypoxic conditions, DCs induce high inflammation while decreasing migration (43). With increased expression of CCL5 (C-C motif chemokine ligand, DCs are able to force granulocyte migration to the infection area, while the ability of DCs to capture antigens is inhibited. Moreover, the expression of TREM1, a hypoxia-inducible gene that encodes a protein that can amplify the immune response, was detected on DCs isolated from the synovium of patients with arthritis (38). With hypoxia promoting glycolysis, the metabolism of DC is reprogramed from oxidative phosphorylation (OXPHOS). Both HIF-1α and mTOR are central regulators of the metabolic switch in DCs and a wide range of downstream targets could facilitate this progress. However, more studies are needed to dissect the role of the complex of mTOR- HIF-1α pathway in DCs (44).

## Discussion

Although the pathogenesis of RA remains to be completely understood, accumulating evidence supports that HIF and hypoxia are vital factors for its pathophysiological characteristics, including inflammation, cartilage damage, angiogenesis, etc. Synovial hypoxia, which can modify the metabolic environment, is linked to some pathogenic processes in RA through direct and indirect effects, for which HIF is an essential factor. Therefore, elucidating the expression of HIFs within RA joints will allow us to have a better understanding of their activation, and the mechanisms by which they affect specific cells to contribute to RA progression. Experimental and clinical data have demonstrated a hypoxia-induced up-regulation of HIF within the synovium. Therefore, HIF is a factor that could promote the inflammation and onset of RA. Moreover, cell metabolism varies with oxygen tension in the microenvironment, possibly through the HIF “switch.” Hypoxia is not a distinctive feature of RA, as it can be found in various diseases, especially in inflammatory autoimmune diseases. Moreover, some studies prove that the consequence of blocking angiogenesis pathways may reduce cellular infiltrates as well as decrease the joint damage. For example, tofacitinib, an inhibitor of JAK1 and JAK3, is able to inhibit HIF-1α signaling resulting in the progression of disease. Otherwise, molecules that promote, rather than inhibit, the activity of PHD2, are more likely to promote the HIF-1α signaling and its proteasomal degradation. Therefore, synovial angiogenesis and leukocyte infiltration are decreased (45). However, the exact function of HIF in autoimmune disease pathogenesis or disease development remains unclear, and whether HIFs play different roles during different stages of disease needs to be clarified.

Overall, further studies will be required to clarify the relationship between the pathophysiology of RA and HIF, in order to discover novel therapies to treat RA or other autoimmune diseases with great therapeutic efficacy and stability following systemic administration.

## Author Contributions

XG and GC wrote and edited the manuscript. All authors contributed to the article and approved the submitted version.

## Conflict of Interest

The authors declare that the research was conducted in the absence of any commercial or financial relationships that could be construed as a potential conflict of interest.
